# Effects of human interleukins in the transgenic gene reporter cell lines IZ-VDRE and IZ-CYP24 designed to assess the transcriptional activity of vitamin D receptor

**DOI:** 10.1371/journal.pone.0193655

**Published:** 2018-02-28

**Authors:** Iveta Bartonkova, Enikoe Kallay, Zdenek Dvorak

**Affiliations:** 1 Department of Cell Biology and Genetics, Faculty of Science, Palacky University, Olomouc, Czech Republic; 2 Department of Pathophysiology and Allergy Research, Medical University of Vienna, Vienna, Austria; Roswell Park Cancer Institute, UNITED STATES

## Abstract

The role of vitamin D receptor (VDR) in immune responses has been broadly studied and it has been shown that activated VDR alters the levels of some interleukins (ILs). In this study, we studied the opposite, i.e. whether 13 selected pro-inflammatory and anti-inflammatory ILs influence the transcriptional activity of human VDR. The experimental models of choice were two human stably transfected gene reporter cell lines IZ-VDRE and IZ-CYP24, which were designed to evaluate the transcriptional activity of VDR. The gene reporter assays revealed inhibition of calcitriol-induced luciferase activity by IL-4 and IL-13, when 1 ng/mL of these two compounds decreased the effect of calcitriol down to 60% of the control value. Consistently, calcitriol-induced expression of *CYP24A1* mRNA was also significantly decreased by IL-4 and IL-13. The expression of *VDR* and *CYP27B1* mRNAs was not influenced by any of the 13 tested ILs. These data suggest possible cross-talk between the VDR signalling pathway and IL-4- and IL-13-mediated cell signalling.

## Introduction

Vitamin D receptor (VDR) is an essential regulator of calcium homeostasis and bone metabolism. It has been shown that calcitriol (one of the D vitamins) also plays crucial roles in other physiological processes including in the induction of cell differentiation, inhibition of cell proliferation, modulation of the immune system and control of other hormonal systems. Hence, any disturbance in the VDR signalling pathway may have severe impact on human health. Given its many roles, the identification of compounds, endogenous or synthetic, that alter the transcriptional activation of VDR is therefore highly relevant.

Modulation of immune responses by VDR has been extensively studied during past decades. Interleukins (ILs) are a group of cytokines that are involved in the communication between immune and inflammatory cells, and 38 ILs have been identified thus far. The first mention of the interaction between 1α,25-dihydroxyvitamin D3 (calcitriol) and the production and functionality of ILs was in the early 1980s [1]. Over the last three decades, the effects of calcitriol on the expression of ILs have been studied in detail [2–5]. However, only a few studies have examined the effects of ILs on the transcriptional activity of VDR. We previously described down-regulation of the VDR-target gene *CYP27B1* by IL-6 and tumour necrosis factor alpha (TNF-α) in the COGA-1A colon cancer cell line, implying that pro-inflammatory cytokines might impair VDR activation, thereby limiting its anti-inflammatory action [6]. Schrumpf *et al*. showed that IL-13 enhances 25-D_3_ (25-monohydroxy vitamin D3)-induced, but not basal, CYP24A1 expression in human primary bronchial epithelial cells. IL-13 was also shown to inhibit basal and 25-D3-induced CYP27B1 expression [7].

In the present study, we examined the effects of 13 pro-inflammatory and anti-inflammatory ILs on the transcriptional activity of VDR. For this purpose, we used the stably transfected human gene reporter cell lines IZ-CYP24 and IZ-VDRE, which were recently developed by our group [8]. Gene reporter assays showed that IL-4 and IL-13 inhibited VDR transcriptional activity, reaching approximately 60% of the calcitriol-induced luciferase signal at concentrations equal or higher than 1 ng/mL in the IZ-CYP24 cell line after 24 h of incubation. Consistent results were obtained for *CYP24A1* mRNA expression. In contrast, the levels of *VDR* and *CYP27B1* mRNA were not influenced by any tested IL, in the presence or absence of calcitriol.

## Materials and methods

### Chemicals and reagents

Thirteen ILs (IL-1α, IL-1β, IL-1RA, IL-2, IL-3, IL-4, IL-5, IL-6, IL-7, IL-8, IL-9, IL-10 and IL-13) were purchased from PeproTech (Rocky Hill, NJ, USA) and reconstituted according to the manufacturer’s instructions. 1α,25-Dihydroxyvitamin D_3_ (calcitriol) was obtained from Toronto Research Centre Inc. (Toronto, Canada). Reporter lysis buffer was obtained from Promega (Hercules, CA, USA). All other chemicals were of the highest quality commercially available.

### Cell lines

The stably transfected human gene reporter cell lines IZ-CYP24 and IZ-VDRE were previously derived from the human colon adenocarcinoma cell line LS180. In brief, LS180 cells were stably transfected with the reporter plasmid CYP24_minP-pNL2.1[Nluc/Hygro] containing a fragment (the base pairs -326/-46) of the human *CYP24A1* promoter (IZ-CYP24 cells) or VDREI3_SV40-pNL2.1[Nluc/Hygro] containing three copies of the VDR response element VDRE-I from the human *CYP24A1* promoter (IZ-VDRE cells) [8]. The transfected cells were cultured in DMEM medium supplemented with 10% charcoal stripped foetal bovine serum, 100 U/mL streptomycin, 100 μg/mL penicillin, 4 mM L-glutamine, 1% non-essential amino acids, and 1 mM sodium pyruvate. Cells were maintained at 37°C and 5% CO_2_ in a humidified incubator.

### Cytotoxicity assay (MTT assay)

Cells (2×10^4^ per well) were seeded in 96-well plates in DMEM supplemented with FBS, and incubated for 24 h. After the incubation with various concentrations of the ILs (1 pg/mL to 100 ng/mL) for 24 h, the culture medium was replaced with medium containing 10% MTT (Sigma Aldrich, Prague, Czech Republic) at a final concentration of 0.3 mg/mL and incubated for an additional 30 min. The absorbance was measured spectrophotometrically at 540 nm using a Tecan Infinite M2000 plate luminometer (Tecan, Männedorf, Switzerland).

### Gene reporter assay

Cells (2×10^4^ per well) were seeded in 96-well plates in DMEM supplemented with FBS, and incubated for 24 h. Then, the cells were treated with the different concentrations of the test ILs (1 pg/mL to 100 ng/mL) in the presence or absence of 50 nM calcitriol. After 24 hours of incubation, the cells were lysed, and luciferase activity was measured using the Tecan Infinite M2000 plate luminometer.

### NanoLuciferase inhibition assay

Cells were treated with 50 nM calcitriol (the model ligand) for 24 h. After incubation, the cells were lysed and cell lysates containing NanoLuciferase were collected. The lysates were mixed with the highest concentration of each tested compound, and luciferase activity was measured using the Tecan Infinite M2000 plate luminometer. A decrease in luciferase activity of less than 15% in the gene reporter assays was considered a non-effect.

### RNA isolation and quantitative reverse transcriptase polymerase chain reaction

Total RNA was isolated using TRI Reagent (Sigma Aldrich, Prague, Czech Republic). Then, cDNA was synthesized from total RNA (1000 ng) using the High-Capacity cDNA Reverse Transcription Kit (Applied Biosystems, Foster City, CA, USA). qRT-PCR was carried out using Power SYBR^®^ Green PCR Master Mix (Applied Biosystems, Foster City, CA, USA) on a StepOne^™^ Plus Real-Time PCR System (Applied Biosystems). *VDR*, *CYP24A1*, *CYP27B1*, and ribosomal protein lateral stalk subunit P0 (*RPLP0*) genes were detected using the primers listed in Table 1. The following PCR program was used: an activation step at 95°C for 10 min, followed by 40 cycles of PCR (95°C for 15 sec; 60°C for 1 min). All measurements were performed in triplicates. The melting curves for the amplified fragments were verified to contain a single peak. Gene expression was normalized to *RPLP0* as a reference gene. Data were processed using the delta-delta C_T_ method and calculated relative to untreated control cells.

**Table 1:** Sequences of the primers used for qRT-PCR.

Primer	Sequence
RPLP0 fwd	5’-CCTCATATCCGGGGGAATGTG-3’
RPLP0 rev	5’-GCAGCAGCTGGCACCTTATTG-3’
VDR fwd	5’-CTTCAGGCGAAGCATGAAGC-3’
VDR rev	5’-CCTTCATCATGCCGATGTCC-3’
CYP24A1 fwd	5’-CAAACCGTGGAAGGCTATC-3’
CYP24A1 rev	5’-AGTCTTCCCCTTCCAGGATCA-3’
CYP27B1 fw	5’-TGGCCCAGATCCTAACACATTT-3’
CYP27B1 rev	5’-GTCCGGGTCTTGGGTCTAACT-3’

### Statistical analyses

All experiments were repeated three times, in three independent consecutive cell passages, and all the measurements were performed in triplicates. Data shown in the graphs are expressed as the mean ± SD. The following statistical analyses were used: Student’s paired t-test (for the statistical differences between two groups, and experiments where a single concentration of test compounds was compared to the control) and one-way analysis of variance (ANOVA) followed by Dunnett’s test (for group comparisons, and experiments where several concentrations of the test compound were used). All analyses were performed using GraphPad Prism version 6.00 for Windows, GraphPad Software, La Jolla, CA, USA (www.graphpad.com). A p-value less than 0.05 was considered to be statistically significant.

## Results

### Effect of ILs on the transcriptional activity of VDR in the human transgenic reporter cell lines IZ-CYP24 and IZ-VDRE

**Fig 1:**
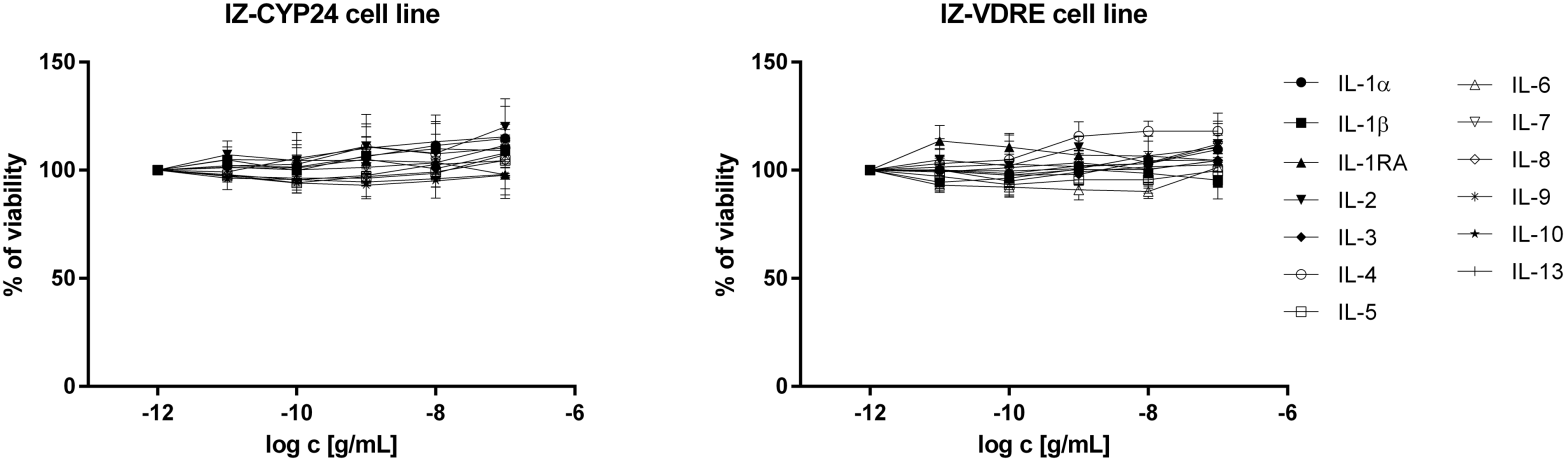
Cytotoxicity of interleukins (ILs) in IZ-CYP24 and IZ-VDRE cells. Cells were treated for 24 h with the tested compounds at concentrations ranging from 1 pg/mL to 100 ng/mL. Incubations were carried out in triplicates. The MTT assay was performed, and the absorbance was measured at 540 nm. Data are the means from three consecutive cell passages and are expressed as a percentage of the viability of the control cells.

The transcriptional activity of VDR was assessed using the stably transfected reporter cell lines IZ-CYP24 and IZ-VDRE. Prior to the gene reporter assays, we examined the effect of ILs on the viability of IZ-CYP24 and IZ-VDRE cells. For this purpose, the MTT assay was performed as described in the Materials and methods section. None of the 13 tested ILs decreased the cell viability (Fig 1). Then, we measured the catalytic activity of NanoLuciferase in IZ-CYP24 and IZ-VDRE cells in the presence or absence of the test ILs. We found that none of the tested ILs had an effect on the catalytic activity of NanoLuciferase (data not shown). We conducted these tests to demonstrate that the ILs do not interfere with the viability of the cells or with the activity of the luciferase, proving that any changes we observe are true effects on the reporter activity or gene expression.

**Fig 2:**
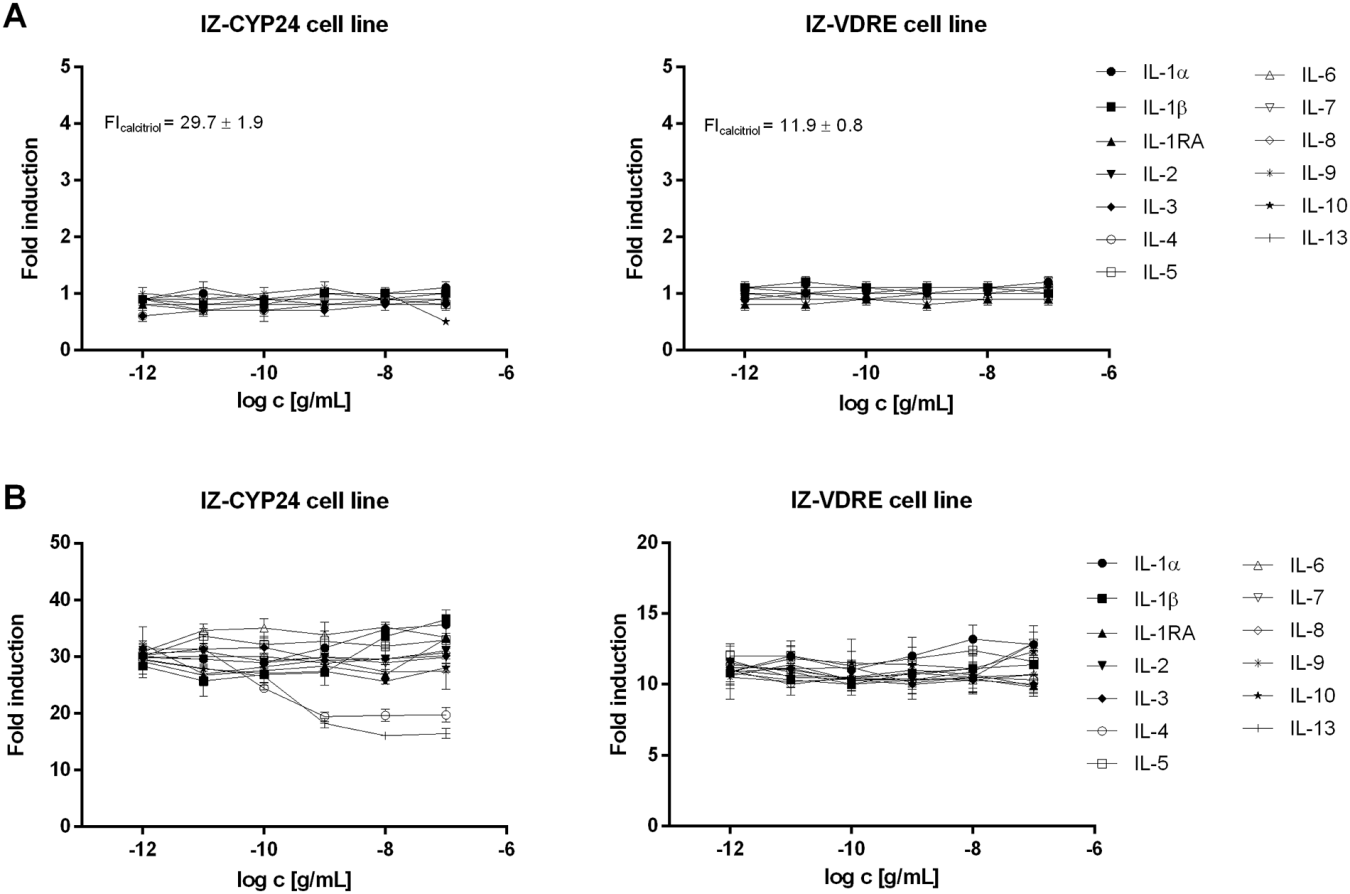
Effects of ILs on the transcriptional activity of VDR in IZ-CYP24 and IZ-VDRE cells. IZ-CYP24 and IZ-VDRE cells were seeded in 96-well plates, stabilized for 16 h, and then incubated for 24 h with the tested ILs, in the presence or absence of calcitriol (50 nM). Treatments were performed in triplicates. The experiments were carried out in three consecutive cell passages. Following the incubations, cells were lysed and luciferase activity was measured. Data are expressed as the fold induction of luciferase activity compared to that in control cells, and shown are the means ± SD from a representative experiment (cell passage) **Panel A:** Incubations in the absence of calcitriol. **Panel B:** Incubations in the presence of calcitriol.

Dose-response analyses were performed in two different experimental layouts, i.e. in the absence or presence of calcitriol. We used calcitriol (50 nM) as the positive control. We observed no induction of luciferase activity by any of the tested ILs in either cell line in the absence of calcitriol (Fig 2A). Calcitriol, the model agonist of VDR, caused an approximate 32-fold and 11-fold induction of VDR-dependent luciferase activity in the IZ-CYP24 and IZ-VDRE cell lines, respectively (Fig 2B). In the presence of calcitriol, IL-4 and IL-13 significantly decreased (to 60% of the initial value) calcitriol-induced luciferase activity in IZ-CYP24 cells but not in IZ-VDRE cells (Fig 2B). Considering that IZ-VDRE cells are primarily responsive to VDR-mediated effects through the VDRE sequences in the promoter, whereas IZ-CYP24 cells contain reporter gene under the control of a 180-bp fragment from the CYP24A1 promoter, we tentatively surmised that these IL-4 and IL-13 effects were VDR-independent. None of the other tested ILs displayed any effect on ligand-induced luciferase activity in either reporter cell line.

### Effects of ILs on the expression of *VDR*, *CYP27B1* and *CYP24A1* mRNAs in IZ-CYP24 and IZ-VDRE cells

We next examined whether the tested ILs influence the expression of the *VDR* gene, as well as the VDR-controlled genes *CYP24A1* and *CYP27B1* in IZ-VDRE and IZ-CYP24 cells. The experimental design and cell incubation were identical to those in the gene reporter assays (*vide supra*), with two incubation times (6 h and 24 h).

**Fig 3:**
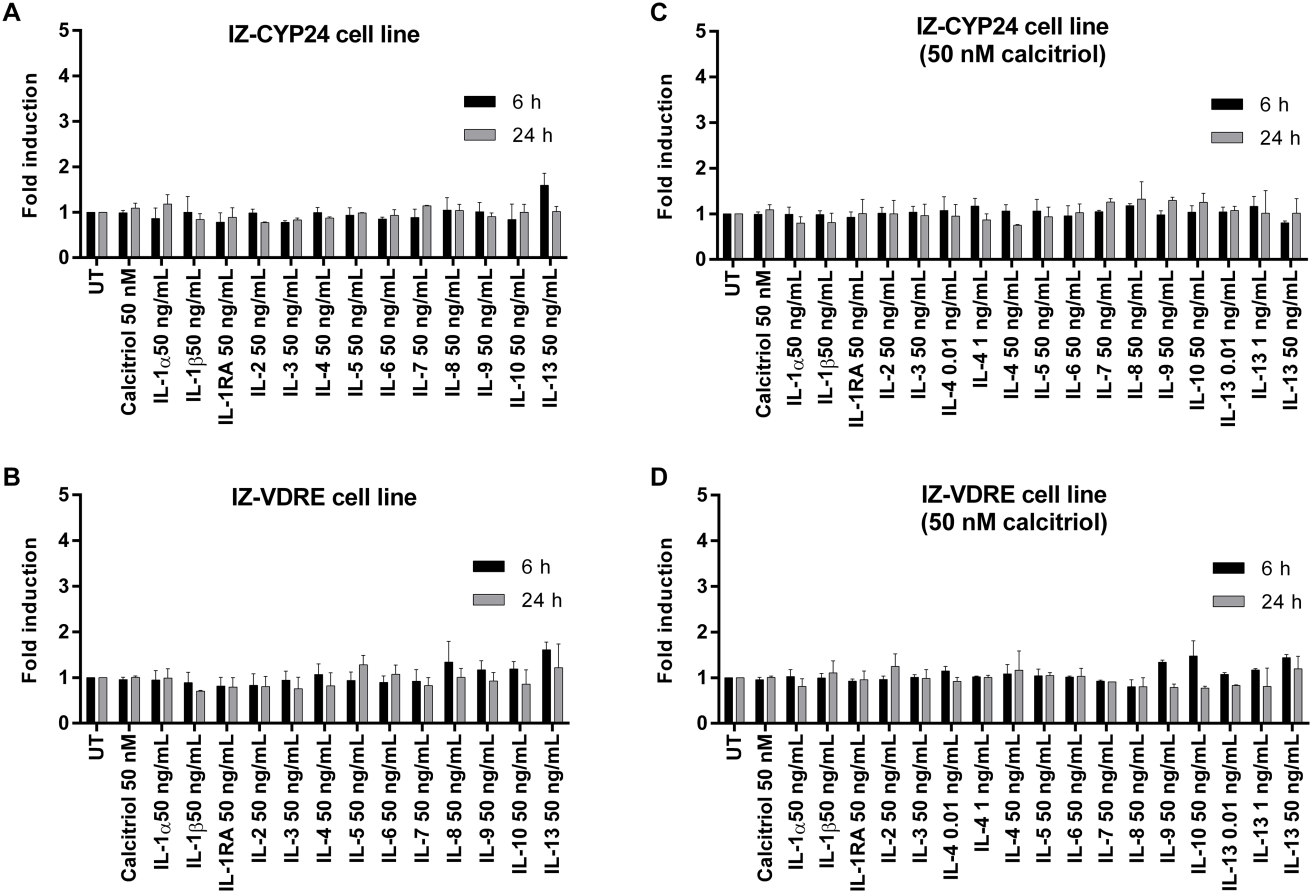
Effects of ILs on the expression of VDR. Cells were seeded in 6-well plates at a density of 7×10^5^ cells per well. Following 16 h of stabilization, cells were incubated with tested ILs for 6 and 24 h in the presence (**C, D**) or absence (**A, B**) of calcitriol (50 nM). The bar graphs show the means ± SD from three consecutive experiments (three independent cell passages), and are expressed as the fold inductions over untreated cells.

**Fig 4:**
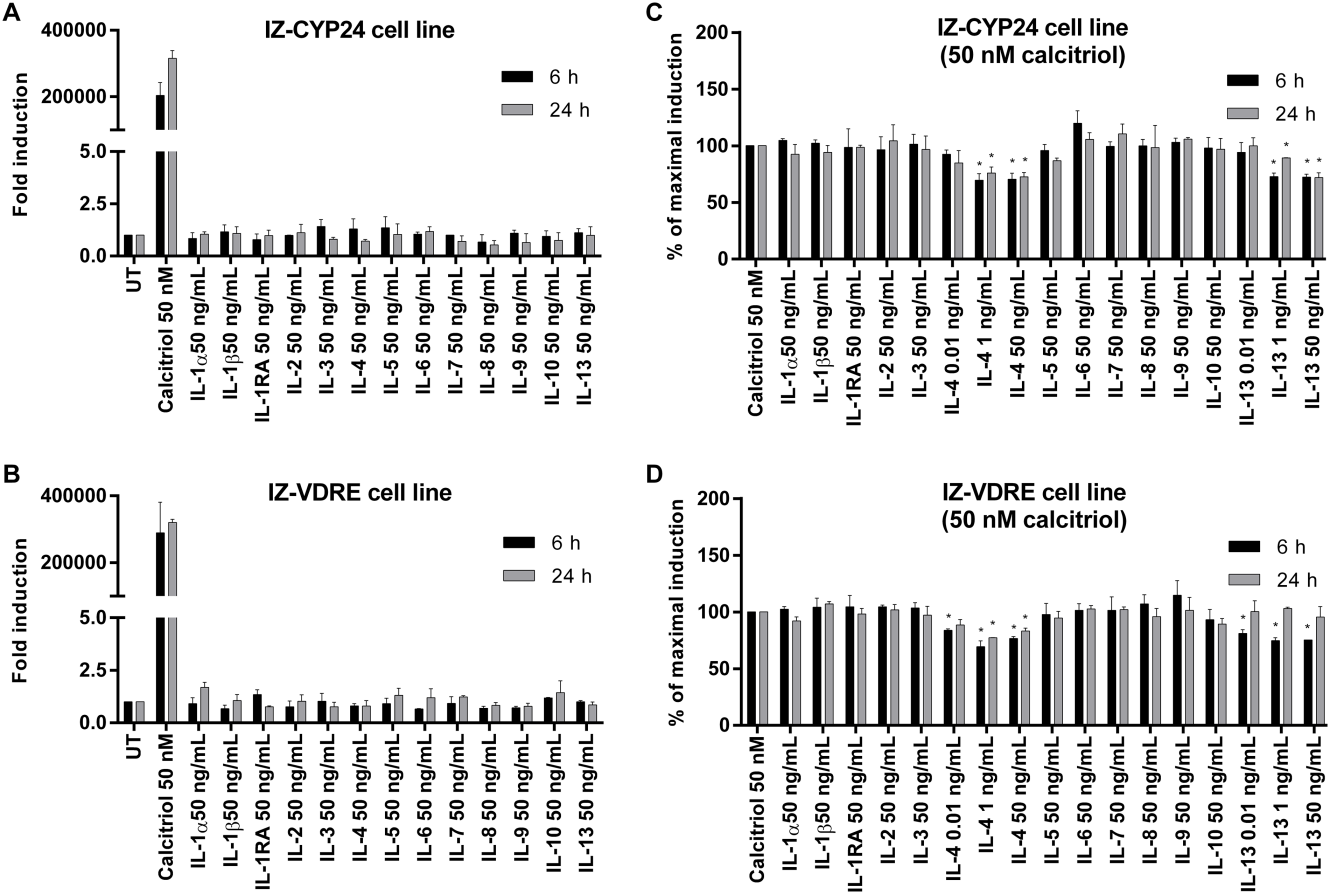
Effects of ILs on the expression of CYP24A1. Cells were seeded in 6-well plates at a density of 7×10^5^ cells per well. Following 16 h of stabilization, cells were incubated with tested ILs for 6 and 24 h in the presence or absence of calcitriol (50 nM). **Panels A and B:** Incubation in the absence of calcitriol. The bar graphs show the means ± SD from three consecutive cell passages, and are expressed as the fold inductions compared to untreated cells. **Panels C and D:** Incubation in the presence of calcitriol. The bar graphs show the means ± SD from three consecutive passages, and are expressed as a percentage of the maximal mRNA expression in the presence of calcitriol alone.

**Fig 5:**
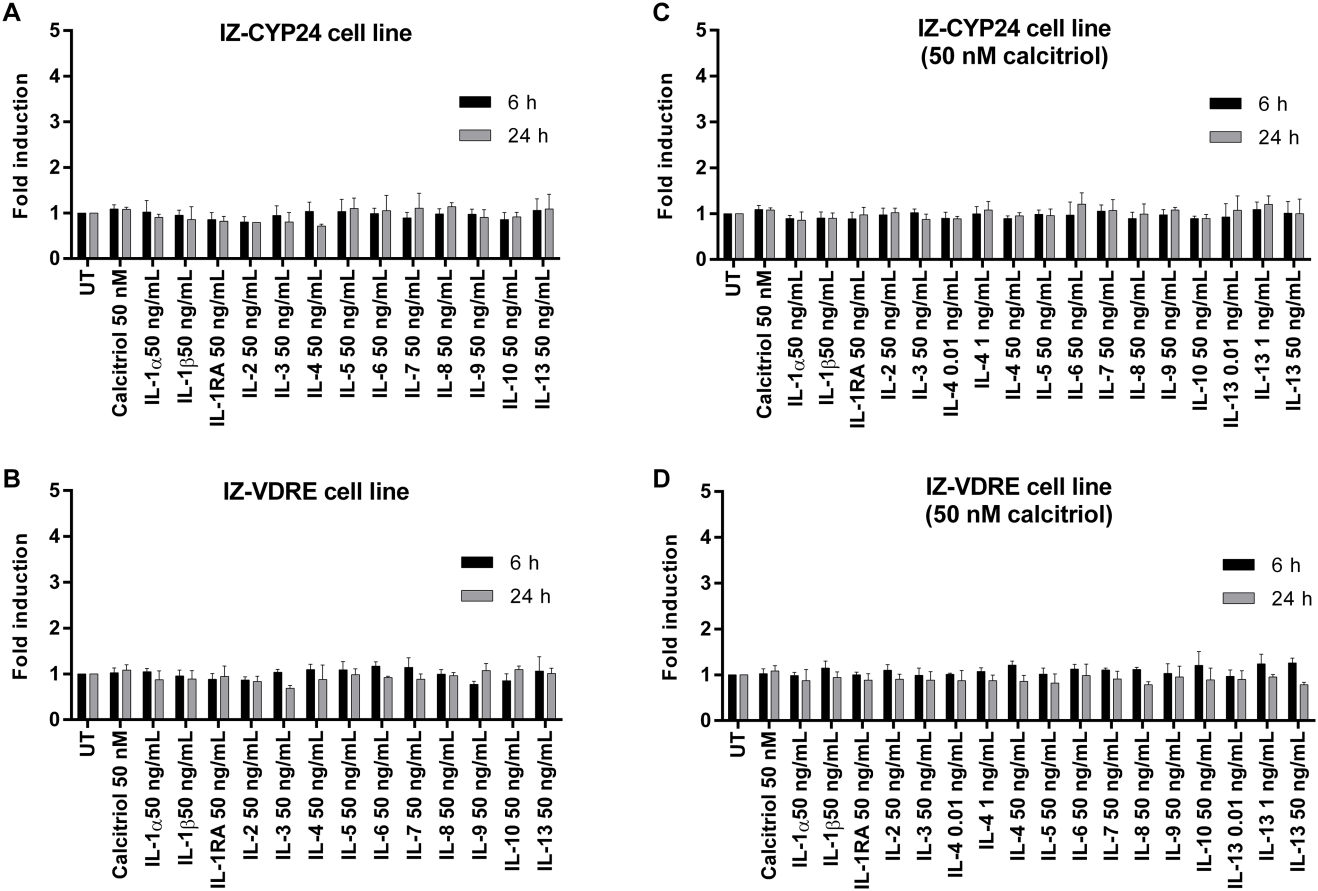
Effects of ILs on the expression of CYP27B1. Cells were seeded in 6-well plates at a density of 7×10^5^ cells per well. Following 16 h of stabilization, cells were incubated with thetested ILs for 6 and 24 h in the presence (**C, D**) or absence (**A, B**) of calcitriol (50 nM). The bar graphs show the means ± SD from three consecutive experiments (three independent cell passages), and are expressed as the fold inductions compared to untreated cells.

Calcitriol drastically induced *CYP24A1* mRNA levels in both IZ-CYP24 and IZ-VDRE cells, yielding fold inductions from 1×10^5^ to 3×10^5^ compared to the levels in untreated cells after 6 h and 24 h of incubation. In contrast, the levels of *CYP27B1* and *VDR* mRNA were not changed by calcitriol treatment at any of the incubation times. Basal mRNA expression of *VDR* (Fig 3A and 3B), *CYP24A1* (Fig 4A and 4B) and *CYP27B1* (Fig 5A and 5B) was not influenced by any of the tested ILs in either cell line, at either incubation time. Furthermore, no changes in *VDR* (Fig 3C and 3D) and *CYP27B1* (Fig 5C and 5D) expression were observed in either cell lines when co-incubated with calcitriol and the tested ILs. In contrast, calcitriol-induced expression of CYP24A1 mRNA was significantly decreased in both cell lines by IL-4 and IL-13 after 6 h of incubation, reaching approximately 70–80% of the level in the presence of calcitriol alone. This effect was maintained after 24 h of incubation for both ILs in IZ-the CYP24 cell line, but only for IL-4 in IZ-VDRE cell line (Fig 4C and 4D). The latter finding is consistent with the data from the reporter gene assays (Fig 2B). However, the PCR data from IZ-VDRE and IZ-CYP24 cells should be similar, because both cell lines were derived from the same parental cell line LS180. Hence, some shift in cell phenotypes during the selection process is anticipated.

## Discussion

ILs are a group of pro-inflammatory and anti-inflammatory cytokines that modulate the responses of the human immune system. Extensive research has been conducted over the past few decades to investigate the roles of VDR in cytokine production. Conversely, ILs were shown to modify the activity of nuclear and steroid receptors. For instance, the transcriptional activity of the glucocorticoid receptor (GR) was inhibited by IL-2 through a mechanism involving signal transducer and activator of transcription 5 (STAT5) [9]. The IL-1, IL-2, IL-4 and IL-6 were shown to modulate the expression and function of GR [10, 11]. In addition, the pro-inflammatory cytokine IL-1α significantly reduced dexamethasone-induced GR-mediated gene transcription in L929 cells by blocking GR translocation to the nucleus [11]. IL-6 was described as a non-steroid activator of the androgen receptor (AR) that functions through a ligand-independent mechanism to induce AR expression [12–15]. IL-6 down-regulated the level of pregnane X receptor and the constitutive androstane receptor [16]. However, information on the effects of ILs on the transcriptional activity of VDR is scarce [6, 7]. IL-13 was reported to increase the expression of CYP27B1 leading to acceleration of the transformation of 25(OH)D_3_ into calcitriol, thereby increasing the expression of CYP24A1 [7]. Similar to our results, IL-6 had no significant effect on VDR, CYP24A1 and CYP27B1 expression in another colon cancer cell line [6].

In the present study, we examined the effects of 13 pro-inflammatory and anti-inflammatory ILs on the transcriptional activity of human VDR, using the stably transfected gene reporter cell lines IZ-CYP24 and IZ-VDRE. Gene reporter assays revealed that IL-4 and IL-13 inhibited calcitriol-induced luciferase activity by 40% in IZ-CYP24 cells but not in IZ-VDRE cells. Because IZ-VDRE cells primarily respond to VDR-mediated effects through the VDRE sequences in the promoter, and IZ-CYP24 cells contain the reporter gene under the control of a 180-bp sequence from the *CYP24A1* promoter that contains binding sites for many other transcriptional factors in addition to VDR, it appears that the effects of IL-4 and IL-13 are independent of VDR. None of the other tested ILs had any significant effect on basal or calcitriol-induced luciferase activity. Consistent with the gene reporter assay results, calcitriol-induced expression of *CYP24A1* mRNA was significantly decreased by IL-4 and IL-13 in both cell lines. In contrast, the expression of *VDR* and *CYP27B1* mRNAs was not influenced by any of the tested ILs in either cell line, regardless of incubation time or the presence of calcitriol. These findings are consistent with our previous results from COGA-1A colon adenocarcinoma cells, where we observed no modulatory effect of IL-6 on *CYP27B1* and *CYP24A1* mRNAs levels. While we the expected significant up-regulation of *CYP24A1* mRNA by calcitriol, we did not observe any change in the expression of *CYP27B1* or *VDR* mRNA in either of the cell lines when incubated with calcitriol. This was unexpected, as there are reports in the literature showing that calcitriol down-regulates CYP27B1 expression through trans-repression [17]. This type of regulation is highly relevant in the kidney where it ensures the tight homeostatic regulation of calcitriol levels through enzymatic activation (CYP27B1) and inactivation (CYP24A1). A plausible explanation for this apparent discrepancy could be the difference in theresponsiveness of the colon cancer-derived cell lines used in the present study, as compared to that of kidney cells. Colon-specific regulation of vitamin D hydroxylases has been described [18, 19], which may account for the specific CYP hydroxylases expression pattern observed in the present study. For these reasons, the negative effects of ILs should be interpreted with caution. The data presented in the current paper imply possible cross-talk between the VDR signalling pathway and cell signalling mediated by IL-4 and IL-13. Moreover, any signalling molecule, including ligands of nuclear receptors or protein molecules, may have cellular off-targets. For instance, there are well-known non-genomic mechanisms of calcitriol action [20]. Important mechanistic aspects in the signalling by nuclear receptors, steroid receptors and xenoreceptors are so called cross-talks between these players. They occur via multiple mechanisms, including shared the ligand, shared response elements, shared the co-activators, regulating-the-regulator (cascade), metabolic feed-back, etc. [21]. For instance, the xenobiotic sensor PXR was found to bind and trans-activate two proximal VDREs in the promotor of CYP24A1, which partly explains drug-induced osteomalacia [22]. Collectively, the physiological relevance of the data in the present study is very complex and follow-up studies are needed to determine the mechanism underlying the effects of interleukins on the VDR pathway.

## Abbreviations

AR: androgen receptor
CYP: cytochrome P450
GR: glucocorticoid receptor
IL: interleukin
MTT: 3-(4,5-dimethyl-2-thiazolyl)-2,5-diphenyl-2H-tetrazolium bromide
PCR: polymerase chain reaction
RPLP0: ribosomal protein lateral stalk subunit P0
STAT: signal transducer and activator of transcription
TNF-α: tumour necrosis factor alpha
VDR: vitamin D receptor
